# King's College Hospital

**Published:** 1902-07-05

**Authors:** 


### Around the Hospitals.

### KING'S COLLEGE HOSPITAL.

### THE YEAR'S WORK.

King's College Hospital has just completed its 63rd year's work. There are 223 beds in 13 wards, one of which, the Pantia Ralli, is endowed to the extent of £6,000. There are, besides, 12 endowed beds and four endowed cots, the remainder being supported by contributions amounting last year to £13,541 14s. 2d. This sum was made up of annual subscriptions, £2,193 5s.; donations, £3,663 8s. 10d.; boxes, £341 9s. 4d.; King Edward's Fund, subscription, £1,000; donation, £350; Hospital Sunday Fund, £1,250; Saturday Fund, £311 15s.; congregational collections (apart from the Sunday Fund), £30 18s. 6d. The income from invested funds amounted to £1,453 5s. 6d.; probationers' fees, £57 15s.; patients' payments, £146 7s.; the David Hughes Fund (1900 and 1901), £2,025; and other receipts, including two charities of £204 and £300, and sale of bottles, £109 15s. 5d. came to £718 10s. This gives £13,541 14s. 2d. as ordinary income. The extraordinary income consisted of legacies, which amounted to £9,526 5s. Among these were £5,000 from Mr. Henry Vaughan, £2,048 from Mr. T. T. Taylor, and £1,000 from Mr. W. Lethbridge. More than half the amount received in legacies has been transferred during the year to the general account.

The total expenditure was higher than usual, being

£20,901 7s. 2d.; and there is a deficit on the year of £2,166 12s. The Prince of Wales's Fund, in 1900, gave a special donation of £250, in the hope that certain articles of food, *i.e.* tea, sugar, and butter, which the patients have been accustomed to provide, should in future be provided free of cost. This, it was calculated, would increase the expenditure by about £400 to £500 a year, but the committee of management quite agreed that it was a right step to take, and would be for the greater comfort of the sick poor. A comparison between the accounts for 1901 and those for 1900 shows that the expenditure on butter, cheese, etc., has increased by £741 5s. 7d., and on grocery by £485 9s. 4d., giving a total of £1,226 14s. 11d. Part of this increase may be accounted for by the increased cost of certain articles of food coming under the head of grocery, but it looks as if the calculations of the committee had fallen short of their actual liability by about half.

The total number of patients under treatment in the hospital was 2,770, of whom 1,033 were discharged cured, 1,029 relieved, 299 unrelieved, and 215 died (of these 59 died within two days of admission). Eighteen patients were found to be dead when admitted. The daily average of beds occupied was 184. In the out-patient department the new cases were 20,471, of which 9,102 were casualties, and 369 poor women were attended in their own homes during confinement.

The urgent need now is for (1) increased out-patient accommodation; (2) special departments, particularly for throat and ear treatment; (3) additional rooms for the nursing and domestic staff, and for the resident medical officers. To enable the Hospital to make use of the latest discoveries, and to maintain its historical position, not only as a curative institution, but in the fore-front of medical and physical science, improved and extended accommodation must, says the report, be provided for many purposes, including electrical and light treatment, and radiography.

The committee earnestly appeal for £75,000 for these purposes.

